# The History of the ABC Proteins in Human Trypanosomiasis Pathogens

**DOI:** 10.3390/pathogens11090988

**Published:** 2022-08-30

**Authors:** Kelli Monteiro da Costa, Raphael do Carmo Valente, Leonardo Marques da Fonseca, Leonardo Freire-de-Lima, Jose Osvaldo Previato, Lucia Mendonça-Previato

**Affiliations:** 1 Laboratório de Glicobiologia, Instituto de Biofísica Carlos Chagas Filho, Universidade Federal do Rio de Janeiro, Rio de Janeiro 21941-902, Brazil; 2 Núcleo de Pesquisa Multidisciplinar em Biologia, Universidade Federal do Rio de Janeiro, Campus Duque de Caxias Prof. Geraldo Cidade, Duque de Caxias 25250-470, Brazil

**Keywords:** ABC transporters, sleeping sickness, chagas disease, *T. brucei*, *T. cruzi*

## Abstract

Human trypanosomiasis affects nearly eight million people worldwide, causing great economic and social impact, mainly in endemic areas. *T. cruzi* and *T. brucei* are protozoan parasites that present efficient mechanisms of immune system evasion, leading to disease chronification. Currently, there is no vaccine, and chemotherapy is effective only in the absence of severe clinical manifestations. Nevertheless, resistant phenotypes to chemotherapy have been described in protozoan parasites, associated with cross-resistance to other chemically unrelated drugs. Multidrug resistance is multifactorial, involving: (i) drug entry, (ii) activation, (iii) metabolism and (iv) efflux pathways. In this context, ABC transporters, initially discovered in resistant tumor cells, have drawn attention in protozoan parasites, owing to their ability to decrease drug accumulation, thus mitigating their toxic effects. The discovery of these transporters in the Trypanosomatidae family started in the 1990s; however, few members were described and functionally characterized. This review contains a brief history of the main ABC transporters involved in resistance that propelled their investigation in *Trypanosoma* species, the main efflux modulators, as well as ABC genes described in *T. cruzi* and *T. brucei* according to the nomenclature HUGO. We hope to convey the importance that ABC transporters play in parasite physiology and chemotherapy resistance.

## 1. Introduction

The *Trypanosoma* genus comprises several species of protozoan parasites of vertebrates that have the following characteristics: a region containing mitochondrial DNA, called the kinetoplast (Kinetoplastida class); a single flagellum (Trypanosomatidae family); and a heteroxenic evolutionary cycle in which it presents itself in one of the evolutionary forms as a trypomastigote in the vertebrate host [1]. Some peculiar characteristics of its cellular biology include: the presence of a flagellar pocket, located close to its single and large mitochondria, where its flagellum comes from, and which is also known for intense endo/exocytosis activity; the presence of glycosomes, which are peroxisome-like organelles that perform glycolysis; a cell membrane supported by a layer of microtubules, highly decorated by specific antigens that are critical for its survival; and proliferation by binary fission, though gene recombination is demonstrated and explains their great genomic complexity [2]. Most species have a heteroxenic life cycle, in which terrestrial vertebrate parasites have part of their cycle in the digestive tract of hematophagous insects and aquatic vertebrate parasites in leeches [3]. Two species of *Trypanosoma* are etiological agents of human disease, causing a great social and economic impact in endemic areas. *T. cruzi* is the etiological agent of American trypanosomiasis or Chagas’ disease and *T. brucei*, the etiological agent of African trypanosomiasis or Sleeping sickness. These two tropical diseases affect roughly eight million people worldwide and are responsible for over 12,000 deaths annually [4]. According to the World Health Organization (WHO), the estimated cost of medical treatment of these diseases exceed 500 million dollars a year (from 2015 to 2030). In addition, infected patients can present morbidities, resulting in damage to the local economy due to absence from work, decreased productivity and medical and chemotherapy expenses [5]. Vector control is the main way to reduce transmission, since there is no vaccine for preventive use in humans. Chemotherapy often has a high cure rate, especially in the early stages of diseases. With the disease progression and, consequently, parasite migration from the blood to the organs of tropism, the limited local bioavailability of the drug leads to a reduction in treatment efficiency. In addition, antitrypanocidal treatment is not able to reverse severe tissue damage caused by the presence of the parasites. Thus, depending on the stage of the disease, chemotherapy can be only reductive. Furthermore, it presents high toxicity leading to adverse effects that can make adherence difficult or prevent patients from continuing treatment [6]. Additionally, resistance to treatment has been reported in these species and results from multifactorial mechanisms, among which we can highlight the presence of ABC transporters [7]. ABC transporters work together to maintain stress response pathways and cellular detoxification and are involved in the multidrug resistance (MDR) phenotype. Although ABC transporters were described in these flagellated protozoa in the early 1990s, little is known about their physiological role and participation in treatment resistance. This review aims to summarize the findings on ABC proteins in these two diseases of epidemiological relevance, emphasizing their participation in chemotherapy resistance.

## 2. ABC Transporters

The ABC (ATP-binding cassette) comprises one of the largest families of proteins in living beings, presenting conserved ABC domains that bind and hydrolyze ATP. This energy is coupled to different cellular processes, including not only the transport of various molecules, but also housekeeping functions [8]. A typical ABC protein presents a nucleotide-binding domain (NBD), also known as an ABC module or ATPase. NBDs are present in a subgroup of the superfamily of P-loop NTPases, which depend on magnesium ions for catalysis. Each NBD has a core of 200 amino acids with two subdomains: the larger RecA-like subdomain, also found in other P-loop ATPases, and α-helical subdomain, unique to ABC proteins. NBDs are mainly identified by three highly conserved motifs: loop A or Walker A, loop P or Walker B and ABC signature or C motif, whose sequences and functions was summarized by Beek et al., 2014 [9]. A typical functional ABC transporter consists of two transmembrane domains (TMD) and two NBDs, and often additional domains connected in different ways [10]. These domains are encoded either as individual proteins or, in varying degrees of fusion, they are expressed as so-called half-size (TMD–NBD or NBD–TMD) or full-size (TMD–NBD–TMD–NBD or NBD–TMD–NBD–TMD) transporters [11]. In the case of half-size transporters, it is assumed that they form dimers or oligomers to carry out the transport of molecules.

All ABC proteins can be divided into three classes by function: classes 1 and 3 are involved with the transport of molecules across biological membranes and class 2 is involved with housekeeping functions [12]. Class 3 contains three types of transporters that are mostly associated with the exclusive function of importing molecules. The three types of ABC importers are found only in prokaryotes. Class 1 is involved with the function of importing and exporting molecules and is present in prokaryotes and eukaryotes [9,13]. To avoid confusion in the nomenclature of ABC members, the scientific community adopted the designation of subfamilies approved by the Human Genome Organization (HUGO). First, seven subfamilies from A to G were created based on sequence homology, phylogenetic relationship and domain organization of eukaryotic ABC proteins (mainly human, mouse and zebrafish genomes). It should be noted that ABCE and ABCF are not transporters; they have two NBDs and are involved in the control of mRNA translation. The enigmatic H subfamily was originally discovered by three genes from Drosophila, and then other genes were discovered in other organisms such as zebrafish and other insects, though they are absent in mammals and plants [14]. Later, subfamily I was created to harbor prokaryotic-like ABCs found in plants that are not present in most animal genomes [15]. In 2020, Thomas and colleagues proposed a new nomenclature for ABC proteins, supported by quantitative analyses using transmembrane helix scores based on the paired structural alignment of TMDs, now organizing its members into seven types of structures. Most eukaryote ABC transporters (subfamily A–G), presenting 6+6 transmembrane helix organization in TMDs, would form types IV and V [16].

One of the most studied ABC proteins is ABCB1, previously known as P-glycoprotein (P-gp or PGP, with P being for permeability) or multidrug resistance protein 1 (MDR1), the first ABC transporter cloned and characterized by its ability to confer resistance to colchicine, the main alkaloid of the poisonous plant meadow saffron, in Chinese hamster ovarian cells [17]. Although ABC was first described in tumor cells, ABCB1 is also expressed in normal tissues, mainly the blood–brain barrier, liver, kidney, intestine, placenta and adrenal, where it participates in the bioavailability of molecules and cellular detoxification [18,19]. ABCB1 can transport a wide variety of substrates, which vary in size, structure and function. Most are weakly amphipathic and relatively hydrophobic, generally containing aromatic rings and a positively charged nitrogen atom [20]. ABCB1 interacts with its substrates through Van der Waals and hydrophobic bonds. Each substrate appears to define a unique niche in the binding cavity by an induced adjustment mechanism [21]. The question remains as to whether the substrate removal would occur in the external aqueous phase (vacuum cleaner model) or whether it would just be translocated from the inner to the outer lipid layer (flippase model) [22]. It is likely that the two models could coexist, according to the size and hydrophobicity of the substrates.

Sixteen years after the discovery of ABCB1, the protein ABCC1 was identified in lung tumor cell lines resistant to anthracyclines, a class of key molecules in neoplastic chemotherapy [23]. The ABCC1 protein was involved in drug transport, being named the Multidrug Resistance Associated Protein 1 (MRP1) [24]. The ABCC1 transporter has a larger structure than the others, thanks to the presence of an extra module with five transmembrane helices, located in the N-terminal portion [25]. The functioning of the transporter is similar to that described for ABCB1 and the extra module does not have a known function; however, it has been proposed that it participates in three-dimensional organization [26]. The ABCC1 transporter shows ubiquitous expression in human tissues [27] and can mediate the cellular efflux of a variety of physiological organic anions, xenobiotics and their metabolites, with many of them co-transported or conjugated to glutathione (GSH), glucuronide or sulfate [28]. GSH is a tripeptide antioxidant and the main non-protein thiol of cells, critical for maintaining the redox state and several cellular processes including drug and free radical detoxification and apoptosis. GSSG is the oxidized dimeric form of GSH, which tends to accumulate in cells under conditions of oxidative stress. Due to their pro-oxidant activities, the maintenance of low GSSG levels and of an appropriate GSH:GSSG ratio are critical for normal cellular functions [29]. As the transport of GSH and GSSG can be carried out by ABCC1, this transporter has an important contribution to redox homeostasis and oxidative stress [27,30].

In 1998, Doyle and colleagues discovered the so-called breast cancer resistance protein (BCRP) and cloned it from a breast cancer cell resistant to doxorubicin, an anthracycline with antitumor and antibiotic properties [31]. In the same year, Allikmets and colleagues characterized a new ABC transporter gene highly expressed in the human placenta and named it ABC placental protein (ABCP) [32]. Additionally, Miyake and co-workers (1998) discovered and cloned two genes designated *MXR1-2*, which codifies mitoxantrone resistance protein 1 and 2, from several human strains resistant to mitoxantrone, another known anticancer drug [33]. The gene sequences and structure of BCRP, ABCP and MXR were almost identical and represented the same transporter, from now on renamed ABCG2, according to the HUGO nomenclature [34]. ABCG2 is a reverse half-size transporter with an NBD–TMD arrangement, which must form homodimers [35] or homotetramers [36] in order to be functional. ABCG2 recognizes a wide variety of molecules, both positively and negatively charged, organic anions and sulfated conjugates [37]. Similar to the other transporters mentioned, it plays an important role in cell detoxification [38]. Interestingly, ABCG2 is highly expressed in human stem cells, this expression being reduced with differentiation. Thus, ABCG2 is a marker for the “side population” phenotype, since the expression of other transporters was not observed in stem cells [39]. The physiological role of ABCG2 in these cells has not yet been fully determined, although it appears to be involved in the transport of perforins [40]. Similar to ABCB1, ABCG2 is also expressed by cells from the placenta, liver, kidney, intestine and blood–brain barrier [41].

### ABC Modulators

Drugs can interact with ABC transporters in several ways. Briefly, an ABC agonist would be a substrate, that is, a drug that is transported by the protein, or an activator of ATPase activity. On the other hand, an ABC antagonist would be a drug capable of inhibiting or reducing drug transport or ATPase activity directly. As the transport function is dependent on ATP hydrolysis, generally the inhibition of the first has a direct impact on the second and vice-versa. An ABC substrate may also be an inhibitor, which prevents the binding of other drugs (competitive inhibition). This type of inhibition will depend on (i) the binding site(s) of the molecule, since ABC transporters have several binding sites for their substrates that may (or may not) overlap, and (ii) the affinity for the transporter [42]. Additionally, drugs can produce a bimodal effect on ATPase activity, where low concentrations stimulate ATPase activity and high concentrations inhibit it, as seen for verapamil (VP) in ABCB1 (see later). Nonetheless, an inhibitor of ATPase activity binds at the catalytic site, acting as a non-competitive inhibitor of transport, a well-known example is vanadate [43]. The association of vanadate with ATP in one of the NBDs results in trapping of the complex and complete abolition of ATPase activity. It is noteworthy that agents such as sodium azide, an inhibitor of the enzyme cytochrome C oxidase, or dinitrophenol and carbonyl cyanide m-chlorophenyl hydrazine (known as CCCP), which inhibit oxidative phosphorylation via decoupling, do not directly interact with ABC transporters but are also able to decrease drug efflux by ATP depletion [44].

Among the mechanisms of modulation of the MDR phenotype, the inhibition of ABCB1 is one of the most observed and was first demonstrated with VP, a calcium channel blocker, in 1981 [45]. In that study, VP increased the *in vivo* and *in vitro* cytotoxicity of leukemia cells resistant to vinca alkaloids. However, low concentrations of VP greatly stimulated the ATPase activity of ABCB1 purified and reconstituted in a lipid bilayer [46]. Ledwitch et al. (2016) demonstrated that the bimodal effect is attributable to the VP binding at two distinct sites in TMD [47]. At low concentrations, VP binds only at the external site, promoting a conformational change (from the open state to the intermediate state), which promotes a partial approximation of NBDs, reducing the energy barrier for ATP hydrolysis and substrate transport (stimulation). At high concentrations, VP occupies both sites and stabilizes the intermediate state, preventing the total approach of NBDs (closed state), reducing VP-induced ATP hydrolysis and substrate transport (inhibition). As VP is a substrate, its inhibition is also competitive, due to occlusion of substrate binding sites [48]. VP is also a known stimulator of ABCC1-mediated GSH transport [49]. Perrotton et al. (2007) demonstrated that VP isomers bind directly to ABCC1 [48]. Only the S isomer interferes with the GSH binding site, increasing the affinity of the transporter for tripeptide and being responsible for its rapid depletion. This effect is known as collateral sensitivity, as cells with an MDR phenotype are more sensitive to the drug, in this case VP, than parental cells. The R isomer competitively inhibits the binding of other drugs, promoting the accumulation of other drugs and reversing the MDR phenotype.

Currently, zosuquidar (known before as LY335979) is a difluoro-cyclopropyl dibenzosuberane derivative and one of most potent and selective third-generation inhibitors of ABCB1, capable of inhibiting *in vitro* and *in vivo* transport at nanomolar concentrations and reversing the MDR phenotype [50]. To date, there is no evidence that zosuquidar is able to modulate the efflux of ABCC1 and ABCG2 [51]. There is a great overlap of ABCB1 and cytochrome P450 isoforms substrates. Zosuquidar is able to inhibit some of these isoforms such as CYP3A; however, it has an affinity that is 60 times lower than ABCB1 [52].

Neyfakh (1988) showed that MDR cells had a reduced accumulation of fluorescent dyes compared to sensitive cells and that the use of inhibitors of the MDR phenotype restored normal accumulation [53]. The results introduced rhodamine 123 (rho 123), a cationic lipophilic dye, as a good fluorescent substrate for ABCB1 for efflux analysis by flow cytometry and fluorescence microscopy, since the use of anti-ABC protein antibodies does not always represent functionality of the transporter. Thus, a simple cell labeling procedure could be used to detect resistant cells and study the MDR phenotype. In the following years, other molecules were described and many ABCB1 modulators were found, as can be seen in the paper from Silva et al. (2015) [20]. 

The efflux activity from ABCC1 transporters can be measured using 5(6)-carboxyfluorescein diacetate (CFDA) dye. It is an acetoxymethyl ester derivative able to cross the plasma membrane efficiently. In the intracellular medium, CFDA is deacetylated by nonspecific esterases. After hydrolysis, the dye gives rise to the fluorescent substrate carboxyfluorescein, which is charged and retained in intact cells and quickly transported by ABCC1, but not by ABCB1 [54]. MK-571 is a potent and selective antagonist of cysteinyl leukotriene receptor 1, synthesized from the receptor backbone and proposed to attenuate the effects of bronchoconstriction induced by leukotriene signaling that occurs in diseases such as asthma [55]. Gekele et al. (1995) showed that MK-571 competitively inhibits the transport of leukotriene LTC_4_ and other organic anions transported by ABCC1, sensitizing MDR cells overexpressing the transporter [56]. It is noteworthy that the ABCC subfamily has 13 members, nine of which are called MRPs because they mediate the MDR phenotype by drug extrusion [57]. Unfortunately, MK-571 has the same effect on C subfamily members that transport LTC_4_, being able to inhibit almost, if not all, MRP members. Nevertheless, it does not affect ABCB1 transport [20,27]. As ABCC1 transport is dependent on GSH, its depletion is able to inhibit/reduce efflux. Depletion of GSH levels can be achieved by treatment with buthionine sulfoximine (BSO), an inhibitor of the GSH biosynthesis pathway, or by thiol alkylating agents such as N-ethylmaleimide (NEM) or iodoacetic acid [58,59]. A list of the modulators of ABCC member transport can be found in the paper by Zhou et al. (2008) [60].

For ABCG2, rho 123 and mitoxantrone are the most commonly used fluorescent substrates [61]. Both are also transported by ABCB1, whose MDR phenotype overlaps considerably with ABCG2 [20,37]. However, two known mutations that occur in the ABCG2 gene during the course of drug selection can alter substrate specificity. For instance, the wild-type ABCG2 gene has an arginine residue at position 482 and cells overexpressing wild-type ABCG2 are capable of extruding mitoxantrone. Nonetheless, cells containing a threonine or glycine residue at the same position are able to transport not only mitoxantrone but also rho 123 [62]. For that reason, mitoxantrone appears to be a better substrate. Fumitremorgin C (FTC) is an organic heteropentacyclic compound that is a prenylated indole alkaloid derived from some fungi such as *Aspergillus fumigatus*. FTC is able to selectively reverse ABCG2-transporter-induced resistance [63,64]. At subtoxic concentrations it has limited, if any, effects on ABCB1 and ABCC transporters. Fumitremorgins have known neurotoxic effects such as tremors and convulsions in animals [65,66], and perhaps for this reason it has not been tested in patients. A tetracyclic analogue of FTC, known as Ko143, proved to be one of the most specific inhibitors among those known for ABCG2 without showing toxicity in mice [67]. However, at higher concentrations it can interact with both ABCB1 and ABCC1 *in vitro* [68]. Szafraniec et al. (2014) and Peña-Solárzono et al. (2017) summarized the other ABCG2 modulators tested [37,69]. The fluorescent substrates and ABC inhibitors are listed in Table 1.

## 3. Human Pathogens of the *Trypanosoma* Genus

### 3.1. T. cruzi

Chagas disease or American trypanosomiasis is a zoonosis caused by the protozoan *T. cruzi*, named in honor of Dr. Oswaldo Cruz. In 1909, the disease was discovered by Dr. Carlos Chagas who published a study with a detailed description of the life cycle of the parasite, vectors and animals that act as hosts, and the clinical characteristics of the disease [70,71]. The WHO estimates that six to eight million individuals are chronically infected with *T. cruzi* in the world, most of them in Latin America, where the disease is endemic [5]. Nevertheless, there is an increasing number of cases in non-endemic regions, such as in European countries, Australia and North America, as a result of migratory processes and alternative routes of disease transmission. The United States has the largest number of infected people outside of the endemic area, with an estimated 300,000 people with Chagas disease living in the country [72].

After an incubation period of approximately two weeks, Chagas disease has an acute phase lasting about two months, characterized by high parasitemia. The acute phase is recognized in few infected individuals, as most cases are asymptomatic or have non-specific symptoms. In the absence of treatment, the infection progresses to the chronic phase [73]. Most individuals remain asymptomatic over the years, without any detectable anatomical and/or physiological alterations, although they present positive serology for *T. cruzi*, characterizing the indeterminate form of Chagas disease. Nonetheless, some infected people may have cardiac, digestive or mixed symptoms, hallmarking the determinate form. These clinical manifestations usually appear between 10 and 30 years after the initial acute infection and affect about 30% of patients. The cardiac form is the most expressive, due to its frequency and severity, causing sudden death in two-thirds of deaths [74,75]. In 1967, nifurtimox was the first oral drug used in the treatment of the acute phase of Chagas disease. Benznidazole is a nitroimidazole derivative synthesized by Wineholt and Liebman in 1972 and the first-choice treatment for Chagas disease thanks to its better efficacy and safety profile [76]. Both drugs are effective in the acute phase and present reduced efficacy with disease progression or presence of severe clinical manifestations [75]. Benznidazole is an oral drug that requires prolonged treatment and presents side effects that are generally dermatological such as itching and rashes, frequent in 30% to 50% of cases, responding well to antihistamines. However, peripheral neuropathy, common in up to 30% of cases, and rare bone marrow depression can develop in patients and lead to immediate discontinuation of treatment [77].

The life cycle of *T. cruzi* is complex and involves morphologically distinct developmental stages, alternating between a triatomine invertebrate vector, also known as a kissing bug, and a mammalian vertebrate host. Epimastigotes are the extracellular replicative forms found in the triatomine’s gut, where they differentiate into infective metacyclic trypomastigotes. Amastigotes are replicative forms found in mammalian nucleated cells that differentiate into bloodstream trypomastigotes [78,79]. *T. cruzi* is a diploid organism and its genome is distributed in pairs of homologous chromosomes that vary in size and number between lineages, indicating high genomic plasticity [80,81]. In 2009, an intraspecific nomenclature for the parasite was proposed by a committee that recognized six genetic lineages or discrete typing units (DTUs), named TcI to TcVI. The strains belonging to the TcI and TcII clade are recognized as pure, while TcV and TcVI are heterozygous hybrids, possibly of the TcII and TcIII parents. TcIII and TcIV are homozygous hybrids and their evolutionary line is still a matter of debate. A seventh DTU was identified as a monophyletic lineage in bats, and is called Tcbat [82]. In 2016, Breniere et al. investigated 137 papers and found a total of 6343 strains of *T. cruzi* distributed in the seven DTUs from 158 host species [83].

In 1987, Filardi and Brener demonstrated the existence of naturally susceptible and resistant strains to the drugs benznidazole and nifurtimox, which could explain the epidemiologic variations in the effectiveness of chemotherapy in infected vertebrate hosts [84]. The first evidence of the involvement of ABC transporters in drug resistance came from the study by Neal et al. (1989) who developed parasites of strain X10, clone 1, resistant to nifurtimox *in vitro* [85]. The nifurtimox-resistant parasites had a half maximal inhibitory concentration (IC_50_) value of 19.6 µM, while the parental parasites had 7 µM. Treatment with VP alone had no effect on the IC_50_ value, but the combination of nifurtimox with 1 µM of VP reduced the IC_50_ of resistant parasites to 2.4 µM.

**Figure 1:**
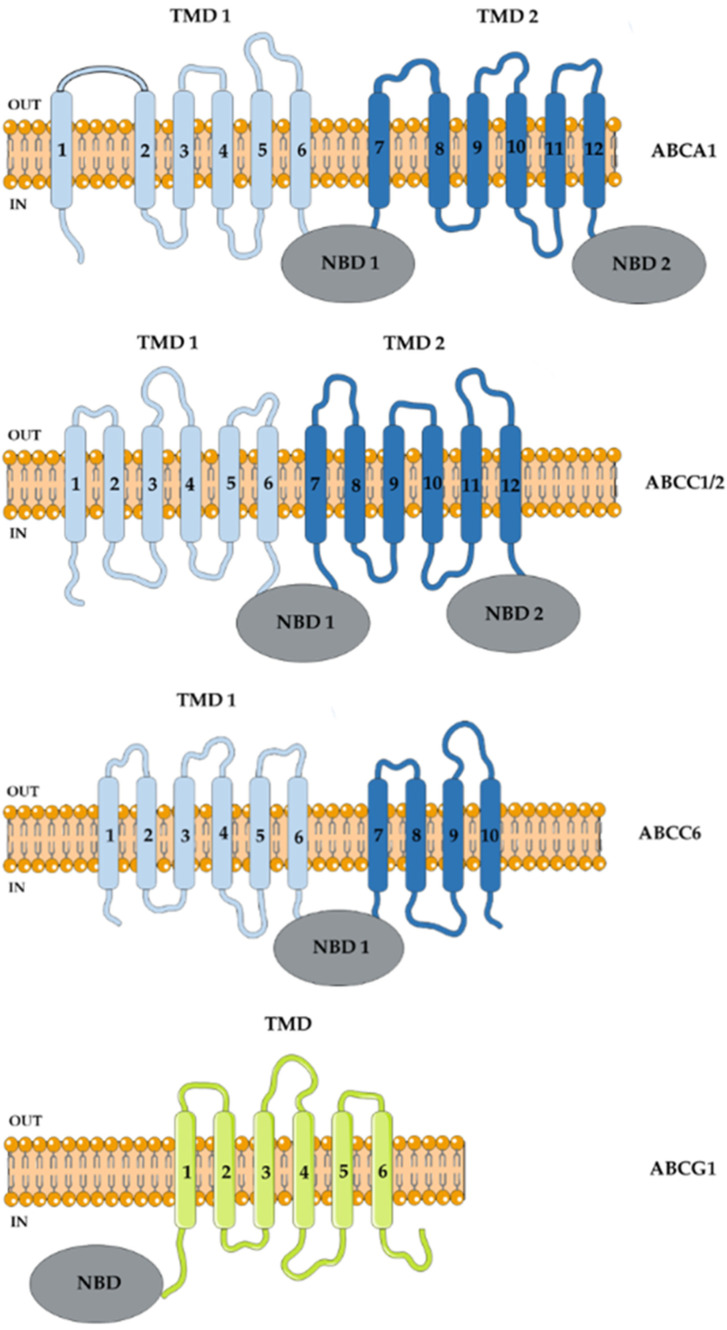
*T. cruzi* ABC proteins. ABC proteins share a nucleotide-binding domain (NBD), displaying conserved sequences among organisms. A typical full-size ABC transporter presents, in addition to the two NBDs, two transmembrane domains, which could be rearranged in direct (TMD–NBD) or reverse (NBD–TMD) order. Half-size transporters have only a TMD and an NBD, assuming that they form dimers or oligomers to transport substrates across membranes. The standardized names of ABC proteins in subfamilies according to HUGO nomenclature are represented by the forth letter, followed by number. For example ABCA1 is subfamily A, member 1.

In 1994, Dallagiovanna et al. were the first to identify sequences homologous to the NBDs from *PGPA* genes of *Leishmania tarentolae* resistant to methotrexate, used to treat certain types of cancer [86], in the Y strain of *T. cruzi* [87]. Using the same probe that identified *LtPGPA*, the authors isolated gene fragments that were sequenced and designated as *TcPgp1* and *TcPgp2*, though the predicted amino acid sequence showed more identity with ABCC1 than ABCB1 [87,88]. Nucleotide sequence analysis of the *TcPgp2* gene revealed an open reading frame (ORF) of 4,602 bp that encoded a 1,534 amino acid polypeptide with an estimated molecular mass of approximately 170 kDa [88]. The structure is similar to that of other ABC proteins, divided into two halves containing six transmembrane helices that make up the TMD and an NBD (Figure 1). The gene is equally transcribed between evolutionary forms, but mRNAs were identified only in the stages of epimastigotes and trypomastigotes, suggesting a post-transcriptional regulation. Due to the difficulty of transfecting *TcPgp2* in epimastigotes of the *T. cruzi* Y strain, Dallagiovanna et al. (1996) performed the transfection in *L. tropica* to evaluate the role of this gene in drug resistance [88]. The transfection resulted in the overexpression of *TcPgp2* transcripts; however, there was no difference in the IC_50_ value in relation to parental cells for different drugs such as benznidazole, nifurtimox, puromycin and daunorubicin. At that time, human ABCC1 showed itself as a transporter of compounds conjugated to GSH, depending on the molecule for efflux of metabolites and drugs. Because of the gene sequence identity of *Leishmania PGPA*, and hence *TcPgp2*, with human *ABCC1* [87], it was suspected that these transporters in protozoa could be transporters of trypanothione [T(SH)_2_], or GSH-conjugated compounds. In trypanosomatids, the main non-protein thiol is T(SH)_2_ that has two GSH molecules linked to a polyamine chain, usually spermidine [89].

Subsequently, Torres et al. (1999) examined the sequence of the *TcPgp1* gene [90]. It has an ORF of 3105 bp that encodes a polypeptide of 1,305 amino acids with an estimated molecular mass of 115,252 Da. The primary structure of the first half of the *TcPgp1* gene is very similar to that of other ABC transporters (Figure 1). Nonetheless, in the second half of the polypeptide chain the 11th and 12th transmembrane helices and the second NBD are absent owing to the insertion of a non-long terminal repeat retrotransposon cDNA with 99% homology to L1Tc, already described in *T. cruzi* [91]. In epimastigotes, the presence of truncated *TcPgp1* transcripts were observed [90], suggesting that they may be functional as homo- or heterodimers, as seen with ABCG2, but this hypothesis has not yet been confirmed.

In an attempt to find a molecular marker associated with benznidazole resistance in *T. cruzi*, Murta et al. (1998) identified polymorphisms in *TcPgp* genes in chemotherapy-sensitive strains [92]. On the other hand, the same group did not observe overexpression or amplification of the *TcPgp1* and *TcPgp2* genes in benznidazole-resistant strains [93], corroborating the data from Torres et al. (1999) [90]. With the completion of *Leishmania* genome sequencing, Leprohon et al. (2006) analyzed and classified 42 ORFs from ABC genes found in the genome of *L. major* and *L. infantum*, according to the HUGO nomenclature [94]. Orthologous ABC genes were found in other trypanosomatids: 27 ORFs in the *T. cruzi* genome and 22 ORFs in *T. brucei*. The gene analysis of *TcPgp1* and *TcPgp2* showed great homology with the *ABCC6* and *ABCC2* genes of *L. major* and *T. brucei*, respectively.

In 2004, Torres and colleagues identified and sequenced a 5253 bp ORF encoding a 1750 amino acid protein with a predicted molecular weight of approximately 200 kDa [95]. The gene was named *TcABC1* and appears to be an ABCA-like transporter, as it shares 33% and 38% amino acid sequence identity with human ABCA1 and ABCA3, and 41% and 47% with LtrABC1.1 and LtrABC1.2 of the ABCA subfamily of *L. tropica*, respectively (Figure 1). This transporter was found to be expressed in the plasma membrane, in the flagellar pocket and in vesicles in the epimastigote and amastigote replicative forms, and it was absent in the trypomastigote infective form. Many members of the ABCA subfamily participate in the trafficking and transport of lipids, including cholesterol that is an important component of cell membranes, regulating fluidity and functionality [96]. Similarly, ABCA from *T. cruzi* appears to be involved in endocytic and exocytic pathways [95].

Heme is an important cofactor necessary for many cellular processes, although trypanosomatids partially or totally lack the enzymes of its biosynthesis pathway [97]. Using the fluorescent heme analog metalloporphyrin, Lara et al. (2007) observed a non-endocytic mechanism of heme accumulation in reservosomes of the Y and Dm28c strains of *T. cruzi*, suggesting specific transmembrane transport [98]. Since ABC transporters are involved with iron transport in bacteria [99] and mammals [100], the authors examined the transport of this analogue in the presence of cyclosporin A (csA) (for inhibition of ABCB1) and indomethacin (for inhibition of ABCC1). The results showed that heme analogue transport was inhibited by csA but not by indomethacin, suggesting that an ABCB1 transporter homologue may be involved [98]. CsA is a cyclic, neutral, lipophilic undecapeptide with immunosuppressive activity produced by a variety of fungi, notably *Tolypocladium inflatum* [101]. Resistance-reversing activity was described by Slater et al. (1986), who showed that csA completely reversed cross-resistance to vinca alkaloids in leukemia strains [102]. Subsequently, Tamai and Safa demonstrated that this resistance reversal was due to the competitive inhibition of csA in the ABCB1 transporter [103]. Indeed, csA is a broad-spectrum modulator because it is able to inhibit the efflux, not only of ABCB1, but also of ABCC1 and ABCG2 [104,105]. Indomethacin is a non-steroidal anti-inflammatory drug, a non-selective inhibitor of cyclooxygenase that catalyze the formation of prostaglandins [106] and an organic anion transport modulator. Therefore, it is able to inhibit ABCC subfamily members [107], ABCB1 [20,108] and other transporters not belonging to the ABC superfamily, such as the organic anion transporter 2 (OAT2) from the solute carrier subfamily [109].

CsA inhibits the proliferation of epimastigote forms of *T. cruzi* *in vitro*, but increases parasitemia and accelerates mortality in infected mice due to its immunosuppressive effects [110]. Non-immunosuppressive derivatives of csA showed an antiparasitic response *in vivo* and *in vitro* [111]. Bua et al. (2008) associated this trypanocidal effect as a result of csA binding to two possible targets: (i) cyclophilins, whose enzymatic activity was sensitive to csA and its derivatives, and (ii) ABCB1, since csA and its derivatives inhibited rho 123 efflux in *T. cruzi* [112]. The authors demonstrated that csA and its derivative F-7-62 reduced the amount of fluorescence in the supernatant of parasites incubated with rho 123, suggesting inhibition of the efflux. The results were similar to controls performed with VP and heat-killed parasites.

Campos and collaborators (2013) induced resistance to the drug benznidazole or to the synthetic compound derived from thiosemicarbazone after 15 passages of the Y strain in the presence of the drugs [113]. The latter was used because of its potent cytotoxic activity in *T. cruzi* [114]. The acquisition of the resistant phenotype coincided with increased levels of transcripts for the *TcPgp1* and *TcPgp2* genes and with cross-resistance to the drugs vinblastine, paclitaxel and daunorubicin, transported by both ABCB1 and ABCC1 [113]. The authors demonstrated the reversal of the MDR phenotype when parasites were treated with the selection drug in the presence of VP or csA, suggesting the participation of an ABC transporter. These results were corroborated by the higher consumption of basal ATP in the presence of selection drugs in resistant strains in relation to parental strains. An efflux of rho 123 inhibited by VP or csA was identified and the authors suggest the participation of an ABCB1 transporter. However, rho 123 can also be transported by ABCG2, and the VP and csA inhibitors exhibit a broad spectrum.

In 2015, a 1998 bp ABC gene with a sequence of 665 amino acids was discovered in the CL Brener hybrid strain [115]. The predicted secondary structure showed that it was a half-size transporter with an NBD–TMD arrangement typical of the ABCG subfamily, then called ABCG1 (Figure 1), with 29% similarity to human ABCG2 and more than 50% with ABCG1 and ABCG2 from *Leishmania* [116]. Naturally resistant strains to benznidazole overexpressed *ABCG1* transcripts and protein compared to sensitive ones. The transfection of the CL Brener strain with a vector containing the *TcABCG1* gene from the naturally resistant strains YuYu and Silvio X10 clone 11 increased the *TcABCG1* transcript and protein level and resulted in an increase in the IC_50_ value for benznidazole in relation to the wild type or vector-transfected strains [115]. The results suggest that the ABCG1 transporter is related to natural resistance to benznidazole in *T. cruzi* strains. Several single nucleotide polymorphisms in the gene sequence [115] and of amino acid variations in the predicted protein sequence [116] were identified in the *TcABCG1* gene, some of which were specific DTUs. Conversely, these differences could not be associated with drug resistance. In an attempt to unravel the mechanisms involved in benznidazole resistance, the same group mapped the *ABCG1* gene in 33 species from seven genera of the Trypanosomatidae family [117]. The authors demonstrated that most benznidazole-resistant species had two copies while *Trypanosoma* species had one copy of the gene per haploid genome. On the other hand, functional analyses were not performed to corroborate the participation of this transporter in drug resistance.

Analysis of the predicted protein sequence for the *TcPgp1* and *Tcpgp2* genes showed high identity and similarity with the *ABCC6* and *ABCC1/2* genes of *T. cruzi*, identified by Leprohon and collaborators by homology to ABC transporter genes of *Leishmania*. Our group identified an ABCC1-like efflux in the Y strain of *T. cruzi*, that was higher in epimastigote than trypomastigote forms. CFDA efflux was shown to be sensitive to: depletion of ATP levels, performed by treatment with sodium azide or iodoacetic acid; depletion of thiol levels, performed by treatment with BSO or NEM; the removal of glucose from the medium during the assay; and also temperature changes [44]. In addition, CFDA efflux was inhibited by MK-571, specific for the ABCC subfamily, and also by probenecid and indomethacin, inhibitors of organic anion transporters in general. GSH, GSSG, ceramide and hemin porphyrin were identified as possible endogenous substrates [118], as well as GSH-conjugated compounds [44], suggesting the participation of this transporter in cellular stress and detoxification pathways. ABCC1-like transporters do not appear to directly transport benznidazole and do not appear to be involved in natural benznidazole resistance, as strains that are naturally sensitive to the drug (CL Brener and Berenice) showed more activity than naturally resistant strains (Y and Colombian) [118]. Da Costa et al. (2021) induced resistance by prolonged exposure to benznidazole, which resulted in a 10-fold increase in the IC_50_ value in the Y strain. This induction of resistance promoted an increase in the ABCC1-like activity, suggesting the participation of the transporter in drug metabolism. The reversal of benznidazole resistance was dependent on the GSH pathway, suggesting that a GSH-conjugated benznidazole compound would be transported. It is noteworthy that there was no ABCB1-like efflux in any of the analyzed *T. cruzi* strains, neither in the epimastigote or trypomastigote forms. Rho 123 efflux was not inhibited, even in the presence of VP, csA and trifluoperazine, three inhibitors capable of inhibiting ABCB1, nor by ATP depletion [44]. *T. cruzi* had a half-size *ABCB1* gene identified in its genome, although the results suggest that there is no functional transport for it. The ABC proteins from *T. cruzi* are listed in Table 2 and displayed in Figure 1.

### 3.2. T. brucei

Human African trypanosomiasis or sleeping sickness is caused by two subspecies of the protozoan *T. brucei*. In 1902, Joseph Everett Dutton identified and named *T. b. gambiense* as the cause of chronic sleeping sickness or the Gambian form [119]. In 1910, John W.W. Stephens and Harold Fanthan identified *T. b. rhodesiense* as the cause of acute sleeping sickness or the Rhodesian form [120]. The disease is unique to sub-Saharan Africa: the Gambian form is found in 24 countries in Central and West Africa, while the Rhodesian form is found in 13 countries in East Africa. Uganda is the only country to have both forms of the disease with non-overlapping regions [121]. The Gambian form accounted for 85% of reported cases in 2020, with the majority occurring in the Democratic Republic of Congo. As a result of disease control and surveillance measures in endemic areas, less than 1000 new cases were reported annually to the WHO since 2018 [122]. WHO data from 2015 estimated that 50–70 thousand people had sleeping sickness [5], with an estimated five million people living in areas of moderate to high risk for sleeping sickness [123]. Currently, sleeping sickness is considered under control by the WHO, which intends to interrupt its transmission by 2030 [5]. On the other hand, it must be considered that the Rhodesian form is a zoonosis, whose main reservoir is cattle [124], thus being difficult to eliminate. In addition, cases of atypical human trypanosomiasis, caused by species not infective to humans, have been reported, indicating an adaptability of species of *Trypanosoma* to new hosts [125].

The incubation period for the disease can range from days to weeks for the Rhodesian form and months to years for the Gambian form. The first stage of the disease is hemolymphatic, in which parasitemia is mainly associated with lymphadenopathy and recurrent episodes of fever. The second stage is meningoencephalitis, in which parasites cross the blood–brain barrier, compromising the central nervous system and resulting in neuropsychiatric disorders, the most characteristic clinical manifestation being dysregulation of the circadian cycle and sleepiness and/or insomnia [126]. *T. b. gambiense* infection is milder and may be asymptomatic or with mild symptoms in the first stage. Patients may live asymptomatically for years or heal spontaneously [127,128]; however, in the vast majority of cases, if left untreated, the patient will die within one to two years after entering the second stage [129]. *T. b. rhodesiense* infection is sporadic, although more severe and with a rapid evolution, leading patients to death within six months [130]. Previously, the oldest trypanocidal drugs suramin, discovered in 1920, and pentamidine, discovered in 1940, were used for the Rhodesian and Gambian forms in the first stage, respectively. In 2019, the nitroimidazole fexinidazole was made available orally as the first-line treatment of both forms in the hemolymphatic stage of the disease and for the meningoencephalitic stage in the absence of severe clinical manifestations [124]. In the second stage, the melaminophenyl arsenical called melarsoprol, discovered in 1949, was used for both forms of disease, although the drug is highly toxic and causes death-associated reactive encephalopathy in up to 10% of patients [131]. For that reason, the ornithine analog eflornithine was introduced in 1990 for the second-stage treatment, being effective only against the Gambian form. In 2009, the nifurtimox–eflornithine combination therapy proved to be more effective, with a lower fatality rate than monotherapy [132].

*T. brucei* is subdivided into five subspecies: *T. b. gambiense* and *T. b. rhodesiense*, which cause human African trypanosomiasis; *T. b. brucei*, which causes animal African trypanosomiasis or nagana; *T. b. evansi*, Surra; and *T. b. equiperdum*, which causes dourine. The subspecies are morphologically similar and the last three do not generally infect humans [124]. Man and cattle are the main reservoirs of *T. b. gambiense* and *T b. rhodesiense*, respectively. The main form of transmission is vectorial and alternative transmission routes are rare. The *T. brucei* life cycle alternates between a hematophagous dipteran vector of the genus *Glossina*, commonly known as tsetse flies, and a mammalian host [126]. Briefly, *T. brucei* has an extracellular life cycle divided into five evolutionary forms. In the vector, the proliferative pro-cyclic forms are found in the intestine; the proliferative epimastigote form and the infective metacyclic trypomastigote form are found in the salivary glands. In mammals, the proliferative slender forms and the non-proliferative stumpy forms are found in the bloodstream. With the exception of the epimastigote forms, the other forms are similar to the trypomastigote forms [133,134].

A stock of *T. b. brucei* isolated from cattle in Somalia showed complete resistance to the trypanocidal drugs diminazene aceturate and quinapyramine sulfate, in addition to reduced sensitivity to homidium chloride, isometamidium, Mel B, and pentamidine isethionate [135]. With the discovery of ABCB1 and its participation in the MDR phenotype, Kaminsky and Zweygarth (1991) tested the effect of VP alone and in combination with trypanocidal drugs on this resistant parasite [136]. Nevertheless, the authors were unable to reverse the resistance phenotype, suggesting that resistance mechanisms in *T. brucei* are not mediated by ABCB1.

**Figure 2:**
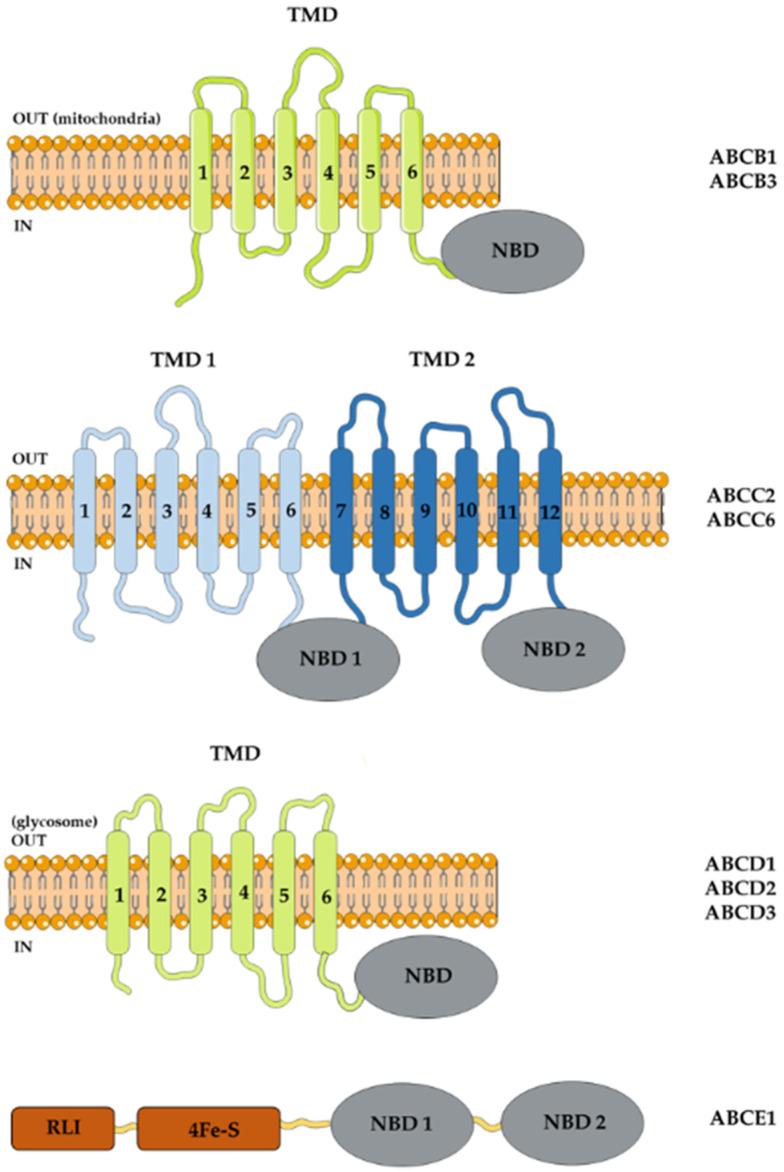
*T. brucei* ABC proteins. ABC proteins share a nucleotide-binding domain (NBD), displaying conserved sequences among organisms. A typical full-size ABC transporter presents, in addition to the two NBDs, two transmembrane domains, which could be rearranged in direct (TMD–NBD) or reverse (NBD–TMD) order. Half-size transporter has only a TMD and an NBD, assuming that they form dimers or oligomers to transport substrates across membranes. The standardized names of ABC members in subfamilies according to the HUGO nomenclature are represented by forth letter, followed by number. For example, ABCB1 is subfamily B, member 1. RLI, L ribonuclease inhibitor domain. 4Fe-S, iron-sulfur cluster binding domain.

Maser and Kaminsky (1998) were the first to identify three first ABC gene sequences, named *TbABC1*, *TbABC2* and *TbABC3* in *T. b. brucei* and *T. b. rhodesiense* [137]. Alignment of the predicted TbABC1 protein sequence showed 65% of residues identical to PGPA from *L. tarentolae*; TbABC2 showed a 57% identity with PGP from *L. donovani*; and TbABC3 showed no homology. The mRNA for the three genes were expressed in pro-cyclic and bloodstream forms of drug-sensitive and resistant parasites. Later, Shahi et al. (2002) demonstrated two predicted proteins of 1581 and 1759 amino acids with genealogy close to *LtPGPA* and *LtPGPE*, belonging to the ABCC subfamily that transports GSH and GSH-conjugated compounds [138]. For that reason, they were called *TbMRPA* and *TbMRPE*, the first containing *TbABC1* discovered by Maser and Kominsky [137]. The authors demonstrated that overexpression of *TbMRPA* or *TbMRPE* in *T. b. brucei* (927 strain) increased resistance to melarsoprol (by 10-fold IC_50_ value) or to suramin (by three-fold IC_50_ value), respectively. TbMRPA was detected by immunofluorescence expressed in the plasma membrane and TbMRPE in the vesicles between the nucleus and the flagellar pocket in the bloodstream forms [138]. In 2006, Lusher and colleagues evaluated resistance to melarsoprol in *T. b. brucei* with MRPA overexpression and/or loss of function of the P2/AT1 aminopurine influx transporter [139]. *MRPA^+^* mutants (IC_50_ = 37.2 nM) were more resistant to melarsen oxide, a melarsoprol metabolite, than *P2/AT1^−^* mutants (IC_50_ = 11.9 nM), with the highest degree of resistance achieved by the double mutant (IC_50_ = 66.1 nM). Phenylarsine oxide is a reducing agent with a high affinity for the thiol radical; it enters the cell by passive diffusion and would be extruded by MRPA [140]. *MRPA^+^* mutants (IC_50_ = 5.6 nM) also showed an increase in drug resistance compared to the parental strain (IC_50_ = 3 nM) and the loss of the P2/AT1 function did not interfere in the process. In contrast, diminazine aceturate, used for the treatment of nagana, enters the cell via the P2/AT1 transporter. Its loss of function raised the IC_50_ value for the drug from 33 nM to 168 nM. *MRPA* overexpression increased the IC_50_ value to 58 nM, though the resistance effect was more pronounced in the double mutant, in which the IC_50_ value reached 264 nM [139]. In the same year, it was demonstrated that overexpression of *MRPA* did not cause resistance in infected mice treated with melarsoprol. As well, gene knockdown by RNAi caused moderate hypersensitivity to melarsoprol and its metabolite melarsen oxide *in vitro*. In addition, overexpression of MRPA was not detected in melarsoprol-resistant clinical samples from sleeping sickness patients [141]. In accordance with the HUGO nomenclature, the ABC genes *MRPA* and *MRPE* are now recognized as *ABCC2* and *ABCC6* (Figure 2) [7].

Besides the transporters, Estevez et al. (2004) found the ribonuclease L inhibitor (RLI) protein, now recognized as ABCE1 in *T. brucei* [142]. The *ABCE1* gene encodes a protein with 631 amino acids and 71 kDa with an RLI metal binding domain, a 4Fe-4S binding domain and two NBDs, and a predicted cytosolic localization (Figure 2). It appears to be involved in protein synthesis, as protein depletion via RNAi resulted in increased riboendonuclease activity and in mRNA levels, in addition to the growth arrest of pro-cyclic and bloodstream forms. Protein overexpression had the opposite effect on riboendonuclease activity and mRNA levels, with a moderate inhibitory effect on cell growth only on bloodstream forms.

In 2006, Yernaux and collaborators discovered three glycosomal ABC transporter (GAT) sequences, named *GAT1-3* in pro-cyclic forms of *T. b. brucei* [143]. Glycosomes are organelles similar to peroxisomes [144]. *GAT1-3* encodes half-size transporters with a TMD–NBD arrangement (Figure 2) with low identity with ABC transporters from mammalian peroxisomes (about 25%) and with colocalization with *T. brucei* glycosomal hexokinase [143]. According to new nomenclature, GAT1-3 are actually the transporters ABCD2, ABCD3 and ABCD1, respectively [7]. ABCD2 and ABCD1 are expressed in both pro-cyclic and bloodstream forms, while ABCD3 is expressed exclusively in bloodstream forms. The results suggest that ABCD2 transports oleoyl-CoA, and possibly other fatty acids, from the cytosol to the glycosomal lumen, as gene silencing results in the accumulation of cellular linoleate, probably due to the presence of an active oleate desaturase [145].

Using the sequence of mitochondrial ABC transporter 1 (*Atm1*) and multidrug resistance-like (*Mdl*) genes from *S. cerevisiae* as the query, Harakova and colleagues (2015) identified homologous genes in the *T. brucei* genome, which were designated *TbAtm* and *TbMdl* [146]. *TbAtm* encodes a protein of approximately 79 kDa and shares about a 40% amino acid identity with the homolog genes *Atm1* from *S. cerevisiae*, *Atm3* from *A. thaliana* and human *ABCB7*. *TbMdl* encodes a protein of a predicted molecular weight of 76 kDa that shows almost a 40% amino acid sequence similarity to the homolog genes *Mdl1* and *Mdl2* of *S. cerevisiae* and human *ABCB10*. Both are mitochondrial ABC transporters: *Atm* is involved in the Fe-S cluster in yeast [147] and in the molybdenum cofactor arrangement in the cytosol in plants [148] while *Mdl* interacts with Fe-S-containing ferrochelatase, an enzyme of the heme biosynthesis pathway in erythroid cells [149]. The sequences *TbAtm* and *TbMdl* correspond to *TbABCB3* and *TbABCB1* (Figure 2), respectively [7], with the TbABCB3 transporter colocalizing with a mitochondrial marker in *T. brucei*. The authors did not propose a precise function of these transporters in the parasite, but the downregulation of *TbABCB3* via RNAi causes a slight reduction in parasite growth, accompanied by a reduction in tRNA thiolation and in the activity of enzymes involved in the Fe-S cluster in the cytosol. Downregulation of *TbABCB1* decreased the mitochondrial heme content, suggesting participation in heme metabolism [146]. The ABC proteins from *T. brucei* are listed in Table 3 displayed in Figure 2.

The MDR phenotype is characterized by cross-resistance to chemically and structurally unrelated drugs. This phenotype is multifactorial, involving the pathways of (i) drug entry, (ii) activation, (iii) metabolism and (iv) efflux. Among the factors involved, the overexpression of ABC transporters is directly related to increased drug efflux and reduced toxic effects within cells. Several protozoan parasites exhibit an MDR phenotype, causing a reduction in the efficacy of chemotherapy. *T. cruzi* and *T. brucei* are not exceptions to the rule, since not only natural but also acquired resistance through prolonged treatment are reported in the literature. ABC transporters were discovered in the context of the MDR phenotype; however, they play important physiological roles, such as the control of cell detoxification, cellular stress and apoptosis. The first ABC genes described in trypanosomatids were in *Leishmania* species in the early 1990s, in parallel with the description of ABCB1/PGP transporter functionality in humans. This comparison led some authors to name the first transporters as PGPs, when in fact their amino acid sequence had more similarity and identity with the ABCC1/MRP transporter. This fact led to some naming confusion, which was standardized with the identification of 42 ABC genes in *Leishmania*, with 27 homologous ABC genes identified in *T. cruzi* and 22 ABC genes in *T. brucei*. At this time, few ABC genes have been characterized in the *Trypanosoma* genus and some are related to chemotherapy resistance. The understanding of how these transporters work in the physiology of the parasite and, consequently, in the acquisition of the resistant phenotype can contribute to the design of new drugs, having these transporters as therapeutic targets, resulting in the improvement of current chemotherapy.

## Figures and Tables

**Table 1:** Fluorescent substrates and ABC inhibitors for efflux analysis.

	ABCB1	ABCC1	ABCG2
FLUORESCENT SUBSTRATE	Rhodamine 123	CFDA	Mitoxantrone
SELECTIVE INHIBITOR	Zosuquidar	MK-571	Ko-143
ATPASE ACTIVITY INHIBITOR	Vanadate		
ATP DEPLETION	Sodium azide, dinitrophenol, CCCP		
THIOL DEPLETION		Iodoacetic acid, N-ethylmaleimide, Buthionine sulfoximine	

CCCP: Carbonyl cyanide m-chlorophenyl hydrazine; MK-571: L-660711, 5-(3-(2-(7-Chloroquinolin-2-yl)ethenyl)phenyl)-8-dimethylcarbamyl-4,6-dithiaoctanoic acid; Ko143: (3S,6S,12aS)-1,2,3,4,6,7,12,12a-Octahydro-9-methoxy-6-(2-methylpropyl)-1,4-dioxopyrazino [1′,2′:1,6]pyrido [3,4-b]indole-3-propanoic acid 1,1-dimethylethyl ester.

**Table 2:** *T. cruzi* ABC proteins.

Nomenclature	*T. cruzi* Gene ID	NCBI Reference Sequences	Location	Putative Protein Length	*T. cruzi* Strain	Alias
ABCA1	TcCLB.504881.50 *	XP_809857	TcChr27	1750	CL Brener Esmeraldo-like	
	TcCLB.510045.20 *	XP_806887	TcChr27	967	CL Brener Non-Esmeraldo-like	
ABCA2/4	TcCLB.507099.80 (a)	XP_817325	TcChr14	1836	CL Brener Esmeraldo-like	
ABCA3/5	TcCLB.504149.20 * (b)	XP_818098	TcChr27	1750	CL Brener Esmeraldo-like	ABC1 [94]
	TcCLB.503573.9 * (c)	XP_803907	TcChr27	967	CL Brener Non-Esmeraldo-like	
ABCA10	TcCLB.510149.80 *	XP_813909	TcChr36	1865	CL Brener Esmeraldo-like	
	TcCLB.506989.30 *	XP_818638	TcChr36	1866	CL Brener Non-Esmeraldo-like	
ABCA11	TcCLB.511725.80	XP_818719	TcChr35	2260	CL Brener Non-Esmeraldo-like	
ABCB1	TcCLB.507093.260	XP_820554	TcChr39	661	CL Brener Esmeraldo-like	
ABCB3	TcCLB.511537.8 *	XP_806158 XP_809384	TcChr35	537	CL Brener Esmeraldo-like	
	TcCLB.511021.70 *	XP_811319	TcChr35	735	CL Brener Non-Esmeraldo-like	
ABCC1/2	TcCLB.506417.10 (d) pseudogene	-	?	1577	CL Brener	Tcpgp2 [86]
ABCC6	TcCLB.509007.99 pseudogene	-	TcChr31	1425	CL Brener Esmeraldo-like	
	TcCLB.507079.30 *	XP_805658	TcChr31	388	CL Brener Esmeraldo-like	
	TcCLB.457101.30 *	XP_805394	?	253	CL Brener	
	TcCLB.508965.14 *	XP_815145	TcChr31	765	CL Brener Non-Esmeraldo-like	Tcpgp1 [86]
ABCC9	TcCLB.510231.29 *	XP_805357	TcChr34	854	CL Brener Esmeraldo-like	
	TcCLB.447255.29 *	XP_803480	TcChr34	220	CL Brener Esmeraldo-like	
	TcCLB.506559.100 *	XP_821792	TcChr34	1472	CL Brener Non-Esmeraldo-like	
ABCD1	TcCLB.506925.530	XP_821597	TcChr39	664	CL Brener Esmeraldo-like	
ABCD2	TcCLB.508927.20 *	XP_804559	TcChr31	674	CL Brener Esmeraldo-like	
	TcCLB.509237.30 *	XP_814630	TcChr31	674	CL Brener Non-Esmeraldo-like	
ABCD3	TcCLB.510431.150	XP_819234	TcChr39	635	CL Brener Esmeraldo-like	
ABCE1	TcCLB.508637.150 *	XP_815243	TcChr10	647	CL Brener Non-Esmeraldo-like	
	TcCLB.511913.9 pseudogene	-	TcChr10	418	CL Brener Non-Esmeraldo-like	
	TcCLB.464879.9 *	XP_802148	TcChr10	339	CL Brener Non-Esmeraldo-like	
ABCF1	TcCLB.504867.20 *	XP_812776	TcChr36	723	CL Brener Esmeraldo-like	
	TcCLB.510943.80 *	XP_817081	TcChr36	723	CL Brener Non-Esmeraldo-like	
ABCF2	TcCLB.508897.30	XP_810886	TcChr40	594	CL Brener Non-Esmeraldo-like	
ABCF3	TcCLB.509105.130	XP_814891	TcChr37	673	CL Brener Non-Esmeraldo-like	
ABCG1/2	TcCLB.506249.70 * (e)	XP_806666	TcChr37	665	CL Brener Esmeraldo-like	
	TcCLB.508231.190 * (f)	XP_818614	TcChr37	665	CL Brener Non-Esmeraldo-like	ABCG1 [114]
ABCG3	TcCLB.506249.70 * (e)	XP_806666	TcChr37	665	CL Brener Esmeraldo-like	
	TcCLB.508231.190 * (f)	XP_818614	TcChr37	665	CL Brener Non-Esmeraldo-like	
ABCG4	TcCLB.506579.10 *	XP_811527	TcChr7	700	CL Brener Esmeraldo-like	
	TcCLB.507241.39 *	XP_806410	TcChr7	290	CL Brener Non-Esmeraldo-like	
ABCG5	TcCLB.504425.70 *	XP_813191	TcChr22	1171	CL Brener Esmeraldo-like	
	TcCLB.509331.200 *	XP_816786	TcChr22	1170	CL Brener Non-Esmeraldo-like	
ABCG6	TcCLB.507681.100	XP_818599	TcChr4	682	CL Brener Non-Esmeraldo-like	
ABCH1	TcCLB.510381.20 *	XP_807302	TcChr27	303	CL Brener Esmeraldo-like	
	TcCLB.506905.40 *	XP_806924	TcChr27	303	CL Brener Non-Esmeraldo-like	
ABCH2	TcCLB.509669.30 *	XP_816112	TcChr36	318	CL Brener Esmeraldo-like	
	TcCLB.509617.80 *	XP_809836	TcChr36	318	CL Brener Non-Esmeraldo-like	
ABCH3	TcCLB.511753.100 *	XP_812902	TcChr32	496	CL Brener Esmeraldo-like	
	TcCLB.511501.30 *	XP_805965	TcChr32	502	CL Brener Non-Esmeraldo-like	
OTHERS	TcCLB.506529.160 *	XP_821943	TcChr6	937	CL Brener Esmeraldo-like	
	TcCLB.510885.70 *	XP_816152	TcChr6	937	CL Brener Non-Esmeraldo-like	
OTHERS	TcCLB.508809.30 *	XP_809200	TcChr23	1241	CL Brener Esmeraldo-like	
	TcCLB.506619.90 *	XP_812627	TcChr23	1241	CL Brener Non-Esmeraldo-like	
OTHERS	TcCLB.507105.70 *	XP_811423	TcChr35	1027	CL Brener Esmeraldo-like	
	TcCLB.506817.20 *	XP_809803	TcChr35	999	CL Brener Non-Esmeraldo-like	

Subfamily names follow the HUGO nomenclature, according to Leprohon et al. (2006) [93]. Gene accession numbers and other information was retrieved from TriTrypDB (tritrypdb.org) and GeneDB database (http://www.genedb.org/Homepage accessed on 10 July 2022). Letters after gene accession numbers indicate genes that present more than one nomenclature due to similar phylogeny to different *L. major* and *T. brucei* genes. Asterisks represent gene fragments of alleles that represent the same *T. cruzi* ORF. (-) represents that the gene ID does not have a protein sequence registered in the NCBI. (?) represents that the gene ID does not have a *T. cruzi* chromosomal location in the TriTrypDB.

**Table 3:** The ABC proteins from *T. brucei*.

Nomenclature	*T. brucei* Gene ID	NCBI Reference Sequences	Location	Putative Protein Length	*T. Brucei* Strain	Alias
ABCA3	Tb927.11.6120	XP_828686.1	TbChr11	1738	brucei TREU927	
ABCA10	Tb927.3.3730	XP_843965.1	TbChr03	1845	brucei TREU927	
ABCB1	Tb927.11.540.2	XP_828146.1	TbChr11	665	brucei TREU927	TbMdl [145]
ABCB3	Tb927.11.16930	XP_829749.1	TbChr11	719	brucei TREU927	TbAtm [145]
ABCC1/2	Tb927.8.2160	XP_847049.1	TbChr08	1581	brucei TREU927	TbMRPA [137]
ABCC6	Tb927.4.4490	XP_844598.1	TbChr04	1759	brucei TREU927	TbMRPE [137]
ABCC9	Tb927.4.2510	XP_844401.1	TbChr04	1503	brucei TREU927	
ABCD1	Tb927.11.1070	XP_828200.1	TbChr11	684	brucei TREU927	TbGAT3 [142]
ABCD2	Tb927.4.4050	XP_844555.1	TbChr04	683	brucei TREU927	TbGAT1 [142]
ABCD3	Tb927.11.3130	XP_828395.1	TbChr11	641	brucei TREU927	TbGAT2 [142]
ABCE1	Tb927.10.1630	XP_822420.1	TbChr10	631	brucei TREU927	TbRLI [141]
ABCF1	Tb927.10.3170	XP_822569.1	TbChr10	723	brucei TREU927	
ABCF2	Tb927.10.15530	XP_828024.1	TbChr10	602	brucei TREU927	
ABCF3	Tb927.10.10880	XP_823306.1	TbChr10	684	brucei TREU927	
ABCG1/2/3	Tb927.10.7700	XP_823004.1	TbChr10	668	brucei TREU927	
ABCG4	Tb927.9.6310	XP_011776526.1	TbChr09	646	brucei TREU927	
ABCG5	Tb927.8.2380	XP_847071.1	TbChr08	1158	brucei TREU927	
ABCG6	Tb927.10.7360	XP_822971.1	TbChr10	646	brucei TREU927	
ABCH3	Tb927.6.2810	XP_845402.1	TbChr06	524	brucei TREU927	
OTHERS	Tb927.1.4420	XP_001219135.1	TbChr01	945	brucei TREU927	
OTHERS	Tb927.2.5410	XP_951709.1	TbChr02	1281	brucei TREU927	
OTHERS	Tb927.2.6130	XP_951745.1	TbChr02	1006	brucei TREU927	

Subfamily names follow the HUGO nomenclature, according to Leprohon et al. (2006) [93]. Gene accession numbers and other information was retrieved from TriTrypDB (tritrypdb.org) and GeneDB database (http://www.genedb.org/Homepage accessed on 10 July 2022).

## Nomenclature

ABC	ATP-binding cassette
ABCA1	Subfamily A, member 1
ABCA3	Subfamily A, member 3
ABCB1	Subfamily B, member 1
ABCB10	Subfamily B, member 10
ABCB3	Subfamily B, member 3
ABCB7	Subfamily B, member 7
ABCC1	Subfamily C, member 1
ABCC2	Subfamily C, member 2
ABCC6	Subfamily C, member 6
ABCD1	Subfamily D, member 1
ABCD2	Subfamily D, member 2
ABCD3	Subfamily D, member 3
ABCE1	Subfamily E, member 1
ABCG1	Subfamily G, member 1
ABCG2	Subfamily G, member 2
ABCP	ABC placental protein
Atm1	Mitochondrial ABC transporter 1
ATP	Adenosine triphosphate
BCRP	Breast cancer resistance protein
BSO	Butionine sulfoximine
CCCP	Carbonyl cyanide m-chlorophenyl hydrazine
CFDA	5(6)-carboxyfluorescein diacetate
CsA	Cyclosporin A
DTU	Discrete typing unit
FTC	Fumitremorgin C
GAT	Glycosomal ABC transporter (GAT)
GPG	P-glycoprotein
GSH	Glutathione
GSSG	Glutathione disulfide
HUGO	Human Genome Organization
IC_50_	Half maximal inhibitory concentration
Ko143	(3S,6S,12aS)-1,2,3,4,6,7,12,12a-Octahydro-9-methoxy-6-(2-methylpropyl)-1,4-dioxopyrazino[1′,2′1,6]pyrido[3,4-b]indole-3-propanoic acid 1,1-dimethylethyl ester
LTC_4_	Leukotriene C4
Mdl	Multidrug resistance-like
MDR	Multidrug resistance
MDR1	Multidrug resistance protein 1 (MDR1)
MK-571	L-660711, 5-(3-(2-(7-Chloroquinolin-2-yl)ethenyl)phenyl)-8-dimethylcarbamyl-4,6-dithiaoctanoic acid
mRNA	Messenger RNA
MRP1	Multidrug resistance protein 1
MXR	Mitoxantrone resistance protein
NBD	Nucleotide-binding domain
NEM	N-ethylmaleimide
OAT	Organic anion transporter
ORF	Open reading frame
Rho 123	Rhodamine 123
RLI	Ribonuclease L inhibitor
RNAi	RNA interference
T(SH)2	Trypanothione
TMD	Transmembrane domain
tRNA	Transporter RNA
VP	Verapamil
WHO	World Health Organization
